# Advancements and challenges of artificial intelligence in climate modeling for sustainable urban planning

**DOI:** 10.3389/frai.2025.1517986

**Published:** 2025-05-20

**Authors:** Teerachai Amnuaylojaroen

**Affiliations:** Atmospheric Pollution and Climate Research Unit, School of Energy and Environment, University of Phayao, Phayao, Thailand

**Keywords:** artificial intelligence (AI), climate modeling, sustainable city planning, machine learning (ML), climate resilience

## Abstract

Artificial Intelligence (AI) is revolutionizing climate modeling by enhancing predictive accuracy, computational efficiency, and multi-source data integration, playing a crucial role in sustainable urban planning. This Mini Review examines recent advancements in machine learning (ML) and deep learning (DL) techniques that improve climate risk assessment, resource optimization, and infrastructure resilience. Despite these innovations, significant challenges persist, including data quality inconsistencies, model interpretability limitations, ethical concerns, and the scalability of AI models across diverse urban contexts. To bridge these gaps, this review highlights key research directions, emphasizing the development of interpretable AI models, robust data governance frameworks, and scalable AI-driven solutions that help climate adaptation. By addressing these challenges, AI-based climate modeling can provide actionable insights for policymakers, urban planners, and researchers fostering climate-resilient and sustainable urban environments.

## 1 Introduction

Climate change compels strategic urban planning to balance growth, sustainability, and societal well-being (Amnuaylojaroen and Chanvichit, 2024). By 2050, estimated 68% of global population lives in cities, making it imperative to develop adaptation strategies that address increasing climate variability and associated risks (Amnuaylojaroen et al., 2024; Parasin and Amnuaylojaroen, 2023). Climate modeling is central to this effort, enabling policymakers to simulate future climate scenarios, assess vulnerabilities, and implement mitigation strategies to enhance urban resilience (Rolnick et al., 2022).

AI, particularly ML and DL, has revolutionized climate modeling by improving data processing, predictive accuracy, and the integration of diverse datasets from satellites, ground-based sensors, and atmospheric monitoring systems. ML algorithms enhance climate models by detecting intricate patterns, refining spatial and temporal resolutions, and producing real-time climate predictions. DL, through multi-layered neural networks, extracts high-level features from complex climate datasets, further refining predictive accuracy and facilitating dynamic climate risk assessments (Lundberg and Lee, 2017; McGovern et al., 2017). These AI-driven enhancements contribute to what is termed “AI-driven climate resilience.” wherein AI-enhanced climate models inform data-driven policy decisions, shaping sustainable urban development.

AI integrates diverse data and enables real-time analysis, yet urban planning assessments remain limited (Brevini, 2021). This review adopts a qualitative literature synthesis, drawing from 2016 to 2025 peer-reviewed sources across AI and climate science. A search from three databases included SCOPUS, Web of Science, and Google Scholar, using keywords such as “AI for climate modeling” and “interpretable ML for urban resilience.” After removing duplicates, 10,616 articles were screened by title and abstract. Of these, due to the broad scope of the searches, strict screening and inclusion criteria were applied to ensure methodological rigor and relevance, 150 articles were reviewed for eligibility. Six case studies were selected based on accessibility, innovation, policy relevance, and geographic diversity. Thematic structuring around data quality, interpretability, and ethics reflects core challenges consistently highlighted in the literature. These themes—data, interpretability, and ethics—frame the review and reflect recurrent priorities in recent AI-climate integration literature.

## 2 AI advancements in climate modeling: enhancing predictive capabilities for sustainable urban planning

**Figure 1:**
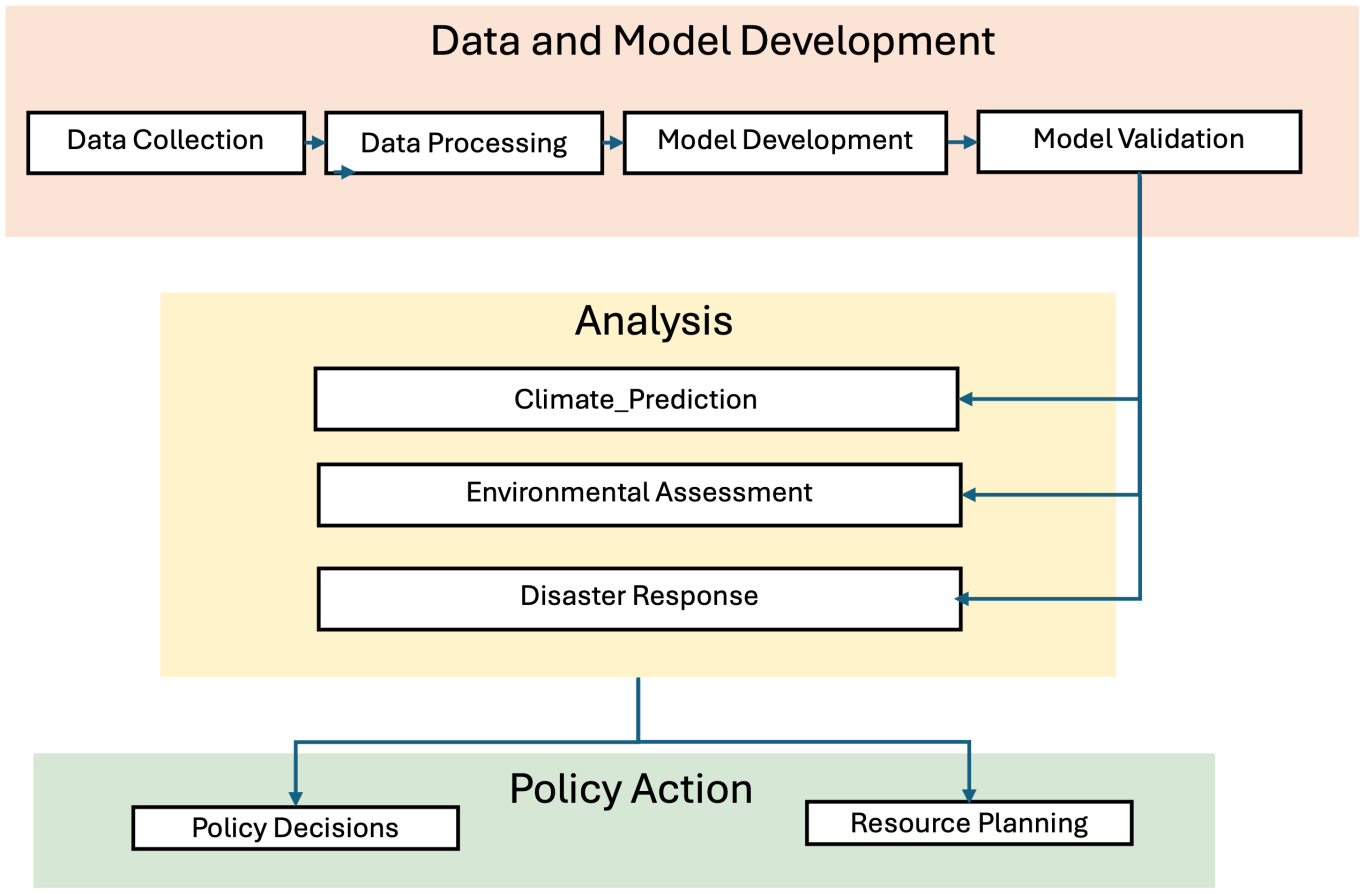
End-to-end AI-driven climate modeling process: from data collection to policy action. Adapted and expanded from Rolnick et al. (2022); figure created by the author.

Artificial intelligence (AI) has transformed climate modeling by improving predictive accuracy, processing efficiency, and data integration. The AI-driven climate modeling process includes three key stages. The first stage, Data and Model Development, involves collecting, preprocessing, and validating climate data from satellites, ground sensors, and historical records, ensuring high data quality for reliable predictions. The second stage, Analysis and Prediction, applies AI models to analyze climate trends, enhance spatial and temporal resolution, and improve climate risk forecasts through advanced machine learning techniques. The final stage, Policy Action and Implementation, translates AI-driven climate insights into strategies for urban planning, disaster mitigation, and resource management, supporting long-term sustainability. Figure 1 illustrates this process, showing how AI enhances climate adaptation strategies and resilience. The schematic highlights AI’s role in improving climate modeling accuracy and informing decision-making. This figure aligns with established AI-based climate modeling frameworks (Rolnick et al., 2022; Karpatne et al., 2017).

### 2.1 Data quality and management

Data quality is crucial for AI-driven climate models due to the complexity of climate data. Sources include ground-based stations, satellites, ocean buoys, and atmospheric sensors have distinct spatial and temporal characteristics. To ensure accuracy, rigorous quality control measures are applied, including outlier detection and gap filling like Interquartile Range (IQR), Z-score normalization, and K-Nearest Neighbors (KNN) imputation (Chandola et al., 2009; Stekhoven and Bühlmann, 2012). These methods enhance data reliability by addressing inconsistencies and missing values, ensuring AI models generate accurate climate predictions.

Homogenization further improves data consistency by adjusting for non-climatic influences through pairwise comparisons with reference datasets (Venema et al., 2012). Standardization aligns data across units, time zones, and coordinate systems, facilitating seamless AI model integration. Additionally, noise reduction techniques such as moving average filters, wavelet transforms, and Kalman filters enhance data clarity (Luo et al., 2019). Cross-validation with independent datasets, such as comparing satellite and ground-based observations, is essential for verifying reliability (Loew et al., 2017). Despite these measures, challenges remain, including data variability and inconsistent spatial–temporal coverage, limiting model scalability and reliability. Furthermore, model interpretability remains an issue, as complex AI models often function as “black boxes,” making it difficult for policymakers to understand predictions. Robust data quality remains a cornerstone for accurate climate predictions, underscoring its importance in producing actionable insights for urban planning and climate resilience. Beyond traditional sources like ground stations and satellites, emerging data streams such as IoT networks, social media, and citizen science are used to improve spatial resolution, capture localized events, and enhance AI-driven climate model responsiveness.

### 2.2 Model interpretability

Beyond technical challenges, AI-driven climate modeling also raises ethical concerns. Bias in training data, lack of transparency, and potential inequities in climate adaptation strategies must be addressed to ensure fair and responsible AI implementation.

Model interpretability is essential for AI adoption in climate science, particularly in policymaking. Techniques like SHapley Additive exPlanations (SHAP) and Local Interpretable Model-Agnostic Explanations (LIME) clarify variable influences, enhancing transparency (Lundberg and Lee, 2017). While ensemble methods, such as Random Forests and Gradient Boosting, enhance predictive performance and robustness, they often increase model complexity, making interpretation more challenging. However, techniques like feature importance ranking in Random Forests provide insights into which climate variables most influence predictions, while model-agnostic methods, such as permutation importance and SHAP values, improve interpretability without compromising accuracy.

Another approach, physics-guided AI, embeds established climate science principles into AI models, ensuring predictions align with known physical laws, thereby enhancing interpretability (Karpatne et al., 2017). While ensemble methods reduce interpretability due to increased complexity, uncertainty quantification techniques—such as confidence intervals and probabilistic predictions—help policymakers assess the reliability of AI-driven climate projections (McGovern et al., 2017). These techniques build trust in AI models, translating predictions into actionable climate strategies.

### 2.3 Ethical considerations

Fairness and transparency are crucial in AI-driven climate modeling. Bias datasets skew projections, harming vulnerable regions by underestimating risks and misallocating resources. Fairness requires diverse datasets and regular bias audits to ensure equitable outcomes (Ghani et al., 2023). Transparency is vital when AI informs urban planning. Clear documentation of data sources, model architectures, and limitations fosters accountability. Compliance with data protection regulations (e.g., GDPR) is essential (EPEUC, 2016). Establishing accountability in AI-driven decisions clarifies responsibilities, ensuring socially responsible and sustainable development (Goodman and Flaxman, 2017).

## 3 Case studies and technical details

To illustrate the practical applications of AI techniques in climate modeling and urban resilience, this section highlights selected case studies that demonstrate how various AI type address the key challenges across different geographic contexts. Case studies underscore AI’s diverse contributions to climate modeling and urban sustainability, with applications spanning forecasting, risk assessment, and environmental monitoring. Table 1 categorizes key AI methods by type—machine learning (ML), deep learning (DL), and hybrid models—and outlines regional implementation challenges. For example, Google DeepMind’s hybrid model, integrating LSTM and CNN, improved wind energy forecasting by 20%, demonstrating the strength of combining temporal and spatial learning in data-rich settings (Buturache and Stancu, 2021). Similarly, the CorrDiff model utilizes deep learning for km-scale atmospheric downscaling, but its reliance on dense datasets limits implementation in regions like Southeast Asia (Mardani et al., 2025). In Southeast Asia, machine learning techniques such as Gradient Boosting and Decision Trees have proven effective for temperature projection refinement, particularly in data-sparse environments (Amnuaylojaroen, 2024). For air pollution forecasting, deep learning models like U-Net are used in urban India to predict PM2.5 trends with high spatial resolution (Rautela and Goyal, 2024). In East Africa, combining Random Forests and SVMs for drought prediction has supported targeted food security interventions (Kogan et al., 2019). Transfer learning using ResNet CNNs has enabled near real-time deforestation monitoring in the Amazon, highlighting the global scalability of deep learning in environmental conservation (Finer et al., 2018).

**Table 1:** Summary of AI techniques applied in climate modeling and urban sustainability.

Method	AI type	Application	Benefits	Limitations	Regional implementation notes	Geographic focus	Data sources	Theme addressed	References
LSTM + CNN	Hybrid (DL)	Wind Energy Prediction	Captures temporal (LSTM) and spatial (CNN) patterns; improves wind forecast	High data needs; computationally intensive	Best in regions with robust energy datasets like the US and Europe	Europe	Monitoring	Data Quality	Buturache and Stancu (2021)
CorrDiff	Deep Learning	High-Resolution Forecasting	Enables km-scale downscaling; improves typhoon and front detection	Demands high-quality training data; calibration needed	Challenges in Southeast Asia due to sparse local observations	Asia	Reanalysis, Observation	Interpretability	Mardani et al. (2025)
Gradient Boosting Machines (GBM)	Machine Learning	Temperature and Air Quality Forecasting	High accuracy with heterogeneous data; robust predictions	Prone to overfitting; sensitive to noise	Effective in Southeast Asia where observational data is mixed	Asia	Global Climate Model, satellite	Data Quality	Amnuaylojaroen (2024)
Decision Trees (DT)	Machine Learning	Temperature Projections	Transparent and easy to interpret	Overfitting on small datasets	Useful in low-resource settings like Laos, Cambodia	Asia	Global Climate Model, Satellite	Data Quality	Amnuaylojaroen (2024)
U-Net Autoencoder	Deep Learning	PM2.5 Forecasting	Extracts spatio-temporal patterns; high-dimensional data capability	Requires labeled data; sensitive to dataset size	Useful in urban India with dense pollution sensors	Asia	Monitoring	Data Quality, Interpretability	Rautela and Goyal (2024)
Support Vector Machines (SVM)	Machine Learning	Drought Prediction	Kernel functions enable flexible modeling	Less efficient with big data; no probability output	Appropriate for regions like East Africa with moderate datasets	Global	Satellite, Monitoring	Data Quality, Ethical Considerations	Kogan et al. (2019)
ResNet CNN (Transfer Learning)	Deep Learning	Deforestation Monitoring	Excellent at feature learning; handles imagery well	High computation; black-box nature	Applicable in Amazon using cloud AI services	South America	Satellite, Global Climate Model, Observation	Data Quality, Interpretability	Finer et al. (2018)

These case studies underscore AI’s versatility in climate modeling, spanning renewable energy forecasting, air quality analysis, drought prediction, and deforestation monitoring. Common AI techniques include hybrid models, ensemble methods, and transfer learning, which leverage satellite imagery, sensor data, and historical climate records. By capturing non-linear relationships and high-dimensional patterns, these approaches enhance predictive accuracy and spatial resolution, providing valuable insights for policymakers and researchers. AI’s integration into climate modeling proves its transformative potential, offering scalable solutions for sustainable urban development and climate resilience.

Despite advancements, AI-driven climate modeling faces key challenges and research gaps. A major challenge is the integration of diverse datasets across regions, as climate data varies in quality, resolution, and availability. This inconsistency limits model reliability, particularly in data-scarce regions like parts of Africa and Southeast Asia. Another concern is the trade-off between model interpretability and accuracy. Deep learning models, while highly accurate, often function as “black boxes,” making it difficult to trust AI-driven climate predictions. Scalability also remains an issue, as AI models must adapt to diverse climatic conditions and efficiently process large datasets without compromising accuracy. Ethical and fairness concerns further highlight the need for inclusive datasets to ensure predictions fairly represent all regions and communities.

Future research could explore reinforcement learning (RL) to optimize urban climate resilience, enabling dynamic adjustments to city infrastructure in response to climate risks. Quantum computing offers new possibilities for simulating complex climate interactions and improving extreme weather predictions. Federated learning enables decentralized AI training with data privacy, while multi-modal systems combining satellite, sensor, and socio-economic data can enhance predictive accuracy for localized impacts. By addressing these challenges and exploring emerging AI technologies, future research can advance climate modeling accuracy, interpretability, and scalability, fostering resilient and adaptive urban environments worldwide.

## 4 Conclusion

This review highlights how AI—particularly ML and DL, and hybrid approaches—has advanced climate modeling for urban sustainability by improving predictive accuracy, enhancing data integration, and enabling real-time decision-making. The analysis of diverse AI techniques reveals varying strengths and applications: ML methods like Gradient Boosting and Decision Trees offer interpretability advantages in regions with limited data infrastructure, while deeper architectures provide superior predictive power in data-rich environments. Implementation success varies significantly by geographical context, with technological readiness and data availability creating disparities between developed and developing regions. While AI offers substantial benefits, critical gaps remain in methodology transparency, data quality, interpretability, and fairness. To maximize impact, future research must prioritize explainable AI models, scalable architectures that function across diverse urban contexts, and ethical design principles that ensure equitable benefits across all communities. Addressing these challenges requires interdisciplinary collaboration among climate scientists, urban planners, and AI specialists to ensure that AI tools serve as scientifically robust and socially equitable instruments for climate resilience, particularly in vulnerable regions facing the most severe climate challenges.
